# MEMS electrochemical angular accelerometer: a paradigm shift for attitude detection and control in rotorcraft UAVs

**DOI:** 10.1038/s41378-026-01326-w

**Published:** 2026-06-04

**Authors:** Maoqi Zhu, Qinghua Liu, Honghao Zhang, Jiesong Yang, Lintao Hu, Wenlang Zhao, Hongmin Jiang, Xiaoye Huo, Yulan Lu, Jian Chen, Yingxun Wang, Deyong Chen, Junbo Wang

**Affiliations:** 1 State Key Laboratory of Transducer Technology, Aerospace Information Research Institute, Chinese Academy of Sciences, Beijing, 100190 China; 2 School of Electronic, Electrical and Communication Engineering, University of Chinese Academy of Sciences, Beijing, 100049 China; 3 School of Automation Science and Electrical Engineering, Beihang University, Beijing, 100191 China

**Keywords:** Electrical and electronic engineering, Engineering

## Abstract

Angular acceleration plays a very critical role for the dynamic control of the accurate attitude estimation of the unmanned aerial vehicles (UAVs), which is conventionally acquired by the differentiation of the gyroscope signals. However, this indirect derivation inherently introduces detrimental phase lags and amplifies noise, thereby compromising the control stability of flight control systems. To address these limitations, this work proposes a MEMS-based electrochemical angular accelerometer (EAA) with high performance, enabling a direct and high-fidelity angular acceleration measurement. Through theoretical modeling and finite element optimization, a compact plate-type electrode structure that enhances hydrodynamic resistance and sensitivity was developed with Glass-on-Silicon (GOS) package. The fabricated device (22 × 22 × 25 mm^3^) achieves a sensitivity of 4.5 V/(rad/s²) and a noise floor of 3.12 × 10^−6^ (rad/s²)/√Hz at 1 Hz, with an ultra-low power consumption of 2.4 mW. While its intrinsic bandwidth is 0.01–0.2 Hz, a compensation circuit extends the -3 dB operational response to 10 Hz. The performance of the EAA was comprehensively validated, ranging from open-loop turntable performance evaluations to flight tests employing a closed-loop incremental nonlinear dynamic inversion (INDI) controller. The results demonstrate that the EAA yields faster command responsiveness and reduced tracking errors when compared to gyroscope-derived estimates. By establishing a robust architecture for direct, low-latency measurement, this work establishes a direct sensing paradigm for high-fidelity angular acceleration measurement in UAV attitude control.

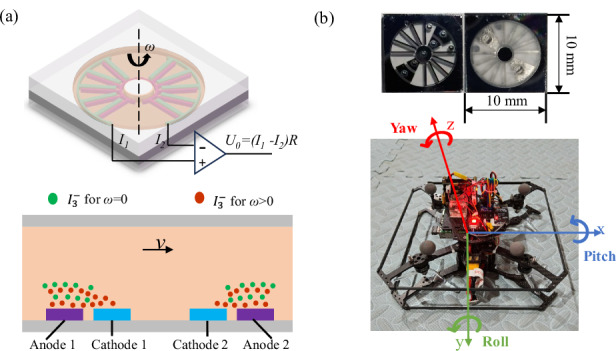

## Introduction

Attitude estimation constitutes a fundamental challenge within the domains of unmanned systems and robotics, where the acquisition of high-fidelity attitude information is vital for vehicle control and trajectory planning^1,2^. In the context of six-degree-of-freedom (6-DoF) flight dynamics, angular motion is an indispensable component. Notably, angular acceleration, serving as a high-order kinematic quantity, directly characterizes transient variations across the pitch, roll, and yaw channels, thereby playing a pivotal role in flight control and navigation. Specifically, angular acceleration functions as a critical input for establishing dynamic models and deriving control laws. Furthermore, under conditions of rapid maneuvering and external disturbances, real-time and reliable angular acceleration data is essential for enhancing the precision and robustness of attitude stabilization^3–5^. Consequently, the precise measurement of angular acceleration remains a focal point of research in the field of inertial navigation.

Current methodologies for acquiring angular acceleration are predominantly stratified into indirect estimation and direct measurement. Indirect acquisition typically involves the temporal differentiation of angular velocity outputs from gyroscope-based inertial measurement units (IMUs). However, despite the ubiquity of MEMS gyroscopes, this differential approach is fundamentally limited by noise amplification. Furthermore, intrinsic sensor imperfections, specifically angle random walk and bias instability, severely deteriorate signal fidelity and induce phase lags, particularly in low-frequency domains^6,7^. An alternative indirect strategy utilizes distributed accelerometer arrays to geometrically reconstruct angular acceleration. While this technique effectively obviates gyroscope-induced drift, it imposes substantial trade-offs regarding system footprint, power consumption, and the stringent requirements for precise spatial alignment and calibration^8^. Concurrently, research efforts have explored “virtual angular accelerometers” based on multi-sensor fusion algorithms^9–11^. Nevertheless, these approaches remain inherently estimative. Their susceptibility to signal noise and insufficient robustness often preclude reliable performance under complex dynamic conditions.

In contrast, direct measurement sensors offer an intrinsic means of acquiring real-time, high-precision angular acceleration data. These devices are generally categorized by their inertial mass into solid-state and liquid-based types. While solid-state variants, such as piezoelectric^12^ and capacitive^13^ sensors, exhibit superior high-frequency response, their low-frequency performance is fundamentally constrained by mechanical structural limitations, rendering them unsuitable for monitoring quasi-static attitude dynamics in aerial vehicles. Conversely, fluid-based sensors utilize a liquid inertial mass to achieve superior performance in the low-frequency domain, characterized by exceptional noise suppression and enhanced sensitivity. This category encompasses microfluidic channel angular accelerometers^14^, liquid-circular angular accelerometers^15–17^, liquid droplet-based accelerometers^18,19^, and electrochemical angular accelerometers (EAAs)^20,21^. Notably, EAAs have attracted significant research attention due to their ultra-low noise floor, excellent low-frequency sensitivity, low power consumption and low cost. However, the deployment of traditional electrochemical sensors on miniaturized UAV platforms is currently impeded by inherent technical bottlenecks, most notably fabrication complexity, substantial packaging footprints, and restricted dynamic range. Consequently, there is a critical need to develop an integrated architecture that maintains electrochemical high-sensitivity and expands bandwidth while minimizing the device footprint.

To address these challenges, this study presents a novel MEMS-based EAA. Through the optimization of the sensitive electrode architecture and microfluidic channel design, the proposed device balances the superior low-frequency characteristics inherent to electrochemical transduction with the compact form factor and high dynamic range demanded by aerial robotics, thus overcoming longstanding bottlenecks in traditional sensors. Additionally, and most distinctively, this study constitutes the first successful implementation of a MEMS EAA as a primary feedback sensor within a rotorcraft’s closed-loop INDI control architecture, transitioning its role from passive motion monitoring to active flight stabilization. Comprehensive comparative analysis against a commercial MEMS IMU (BMI055) substantiates the EAA’s superior performance advantages, including significantly attenuated command tracking error and faster transient response time. These results establish the device as a critical component for next-generation attitude control system.

## Materials and methods

### Working principle

**Fig. 1:**
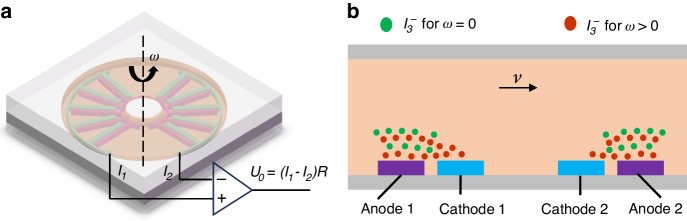
Structure and working principle of EAA. **a** Schematic of the basic structure of EAA. **b** The structure of the four-electrode and the active species in the electrolyte solution are presented, where active species are created near anodes and reduced near cathodes. In the absence of external signal (*v* = 0), the concentration gradient between the cathodes and anodes remains steady; however, when external signals are applied (*v* > 0), it becomes unstable, producing an electrical output

Analogous to conventional inertial sensors, the operational dynamics of the proposed EAA can be modeled through a transfer function comprising two distinct subsystems (mechanical subsystem and electrochemical subsystem). The former acts as a fluidic interface, converting external angular excitation into the convective flow of the electrolyte, while the latter functions as a transduction element, transforming these hydrodynamic signals into measurable electrical currents. As depicted in Fig. 1a, the device architecture integrates three critical components: a microelectrode sensing unit, a functional electrolyte, and a hermetically sealed toroidal housing. The sensing unit features a symmetric electrode array configured in an ACAC (Anode-Cathode-Anode-Cathode) or CACA sequence, positioned centrally within the annular flow channel. Based on empirical values, a mixed solution of 2 mol/L I₂ and 0.02 mol/L KI is selected as the electrolyte.

The fundamental working principle is illustrated in Fig. 1b. A DC bias voltage of 0.3 V is applied across each anode-cathode pair, driving reversible redox reactions at the microelectrode interfaces^22^:

1 $$Anode:3{I}^{-}-2{e}^{-}\to {{I}_{3}}^{-}$$

2 $$Cathode:{{I}_{3}}^{-}+2{e}^{-}\to 3{I}^{-}$$

Given that the concentration of molecular iodine (I₂) is significantly lower than that of iodide ions (I⁻), the concentration of triiodide ions (I₃⁻) serves as the active species. This setup establishes a distinct concentration gradient, where the concentration of active species is approximately 0.02 mol/L near the anode and approaches zero at the cathode surface. In the quiescent state (steady-state conditions), ion transport is governed primarily by diffusion and migration, maintaining a dynamic equilibrium. Consequently, the ion fluxes at the symmetric cathodes are identical, resulting in balanced electrode currents (*I*₁ ≈ *I*₂), and a null differential output voltage (*U*_out_ = 0 V).

Upon the application of an external angular acceleration, the inertial lag of the electrolyte mass induces a relative flow opposite to the direction of excitation. This convective transport disrupts the steady-state concentration profile, increasing the ion flux at one cathode while decreasing it at the other. The resulting asymmetry in electrode currents (*I*₁ ≠ *I*₂) generates a differential output signal (*U*_out_ > 0 V) that correlates directly with the input acceleration frequency and magnitude. Governed by Faraday’s law of electrolysis, these current variations are processed by a differential amplifier circuit, which converts the current signals into voltage while effectively suppressing common-mode interference. Furthermore, a custom compensation circuit is implemented to extend the operational bandwidth and enhance high-frequency fidelity^23^.

### Mathematical model

The mechanical subsystem can be approximated as a mass–damper system, which is subject to inertial force, hydrodynamic resistance, and external angular acceleration^24^. The damping force originates from the viscous resistance of the electrolyte. Based on Newton’s second law, the relationship between the electrolyte flow velocity v inside the annular channel and the external tangential acceleration ${a}_{ex}$ can be derived as:

3 $$m\frac{dv}{dt}+{R}_{h}{{S}_{{\rm{ch}}}}^{2}v=-m{a}_{ex}$$

where *m* is the fluid mass, ${R}_{h}$ is the hydrodynamic resistance, and ${S}_{{\rm{ch}}}$ denotes the cross-sectional area of the annular flow channel. Applying a Laplace transform yields the transfer function of the mechanical subsystem:

4 $${H}_{mech}(s)=\frac{v(s)}{{a}_{ex}(s)}=\frac{1}{s+{\omega }_{h}}$$

*Here*, ${\omega }_{h}$ *is the corner frequency, representing the effective damping of the system, expressed as:*

5 $${\omega }_{h}=\frac{{R}_{h}{S}_{ch}}{\rho \cdot 2\pi r}$$

This transfer function indicates that the mechanical subsystem behaves as a first-order inertial system with low-pass characteristics. The cutoff frequency is determined by the annular channel cross-sectional area (${S}_{{\rm{ch}}}$), the channel radius ($r$), fluid density ($\rho$), and flow resistance (${R}_{h}$). Thus, the channel cross- section and radius are the dominant parameters for controlling the damping ratio.

The electrochemical subsystem is primarily described by the Nernst–Planck equation^25^, which governs the transport of active species under migration, diffusion, and convection:

6 $${N}_{{I}_{3}^{-}}=-D\nabla C-\frac{zF}{RT}/DC\nabla \varPhi +Cv$$

Specifically, migration is neglected due to the high ionic conductivity of the supporting electrolyte. The electrode kinetics are assumed to be infinitely fast, implying that the Nernst equation applies at the electrode surfaces. No-slip boundary conditions are imposed at both the electrode surfaces and the channel walls, and the flow is assumed to be fully developed laminar. By introducing the assumption of ideal electrode boundaries and applying linearization, a simplified mathematical model of the electrochemical subsystem can be established^26^. The resulting transfer function can be expressed as:

7 $$\begin{array}{c}|{H}_{ec}(s)|=|\frac{i(s)}{v(s)}|\propto \frac{1}{\sqrt{1+{\omega }^{2}/{{\omega }_{{\rm{ec}}}}^{2}}}\\ {\omega }_{ec}=D/{d}^{2}\end{array}$$

where ${\omega }_{{\rm{ec}}}$ is the characteristic frequency of the electrochemical process, and *d* is the equivalent diffusion distance.

This subsystem can be approximated as a combination of a proportional element and a first-order inertial element. The gain factor depends on carrier concentration, cathode surface area, and electrode spacing.

Accordingly, the overall transfer function of the EAA can be represented as the product of the two subsystems:

8 $$H(s)=\frac{{U}_{out}(s)}{{a}_{ex}(s)}={H}_{mech}(s)\cdot {H}_{ec}(s)\cdot R$$

where R denotes the load resistance in the external circuit.

### Finite element simulation

**Fig. 2:**
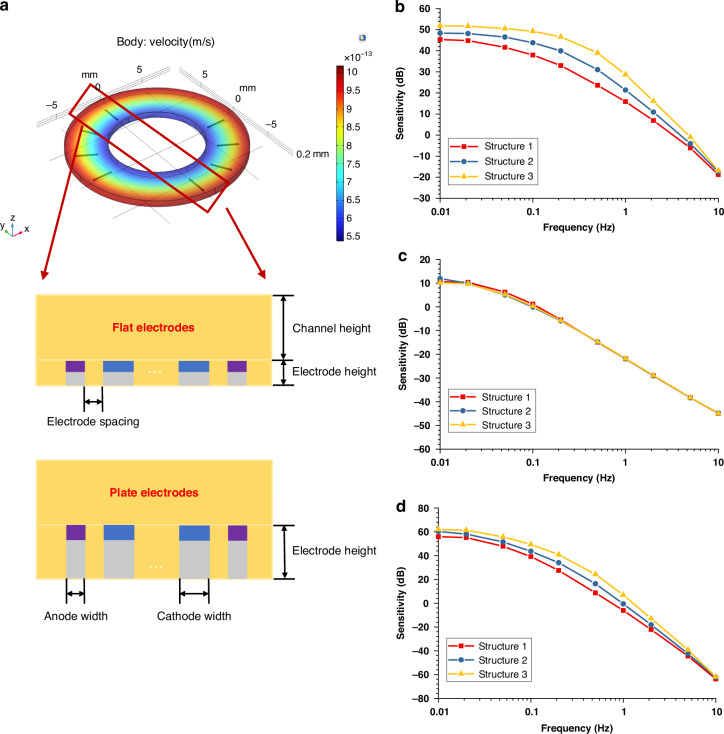
Finite element simulation of plate-type electrode EAA. **a** 3D model and electrode schematics, **b** mechanical, **c** electrochemical, and **d** overall system frequency responses

Given that the EAA’s transduction mechanism involves the intricate coupling of multiple physical domains, specifically fluid dynamics and electrochemistry, a simplified three-dimensional model was established and solved using the COMSOL Multiphysics environment. This simulation served to verify the accuracy of the underlying analytical derivations. The geometry of the sensing unit, particularly the microelectrode configuration, critically dictates the overall performance characteristics of the device. In this study, a novel integrated sensing structure featuring plate-type electrodes was proposed and rigorously compared against a conventional design employing planar electrodes, as illustrated in Fig. 2a. In the model, the annular flow channel is fully occupied by the electrolyte solution, with the anode and cathode represented by distinct colors (typically purple and blue, respectively). Essential physical properties of the electrolyte (density and dynamic viscosity) and the geometric dimensions of the annular channel (e.g., inner and outer radii) are meticulously detailed in Table 1. Furthermore, the design parameters for the plate-type electrodes are summarized in Table 2, where an electrode height of zero corresponds to the conventional planar configuration.

**Table 1:** Material/structure parameters in the simulation

Electrolyte		Channel geometry	
Density	1410 kg/m^3^	External diameter	8.5 mm
Dynamic viscosity	2.28 × 10^−3^ Pa·s	Internal diameter	4.5 mm
Conductivity	1 × 10^3^ S/m	Height	0.5 mm
Concentration of *I*^*-*^	2000 mol/m^3^	\	\
Concentration of *I₃⁻*	20 mol/m^3^	\	\

**Table 2:** Key parameters of sensitive electrodes

	Cathode width (μm)	Anode width (μm)	Electrode spacing (μm)	Electrode height (μm)
Structure 1	200	50	50	0
Structure 2	200	50	50	100
Structure 3	200	50	50	200

The simulation framework employed a bidirectional coupling between mechanical and electrochemical domains to rigorously capture the device physics. The mechanical response was resolved using the Rotational Machinery and Laminar Flow modules, subjected to a harmonic axial angular velocity of 10^-4 ^rad/s across a frequency sweep of 0.01–10 Hz to induce inertial fluid motion within the toroidal cavity. Concurrently, the electrochemical behavior was modeled through the Tertiary Current Distribution interface, which calculated the transient electrode reaction currents governed by the fluidic excitation. A dense boundary layer mesh was applied at electrode edges and fluid-solid interfaces to accurately capture concentration and velocity gradients. Boundary conditions were set as active electrochemical reaction boundaries for electrodes and no-slip boundaries for channel walls to simulate realistic hydrodynamic conditions.

Simulations were performed via time-domain transient analysis in COMSOL, and the resulting data were post-processed using Fast Fourier Transform in MATLAB to obtain frequency response characteristics. As depicted in Fig. 2b, the mechanical subsystem exhibits a positive correlation between response amplitude and electrode height. This enhancement is attributed to the plate electrodes expanding the fluid storage volume, thereby augmenting both the effective inertial mass of the electrolyte and the hydrodynamic resistance within the channel. Conversely, the electrochemical subsystem shows negligible sensitivity to geometric variations (Fig. 2c), indicating that electrode height exerts minimal influence on the intrinsic reaction kinetics. Ultimately, the synthesized system response (Fig. 2d) confirms that the proposed EAA with plate-type electrodes achieves enhanced sensitivity and a broader operational bandwidth compared to its planar-electrode counterpart^27,28^.

### Fabrication

**Fig. 3:**
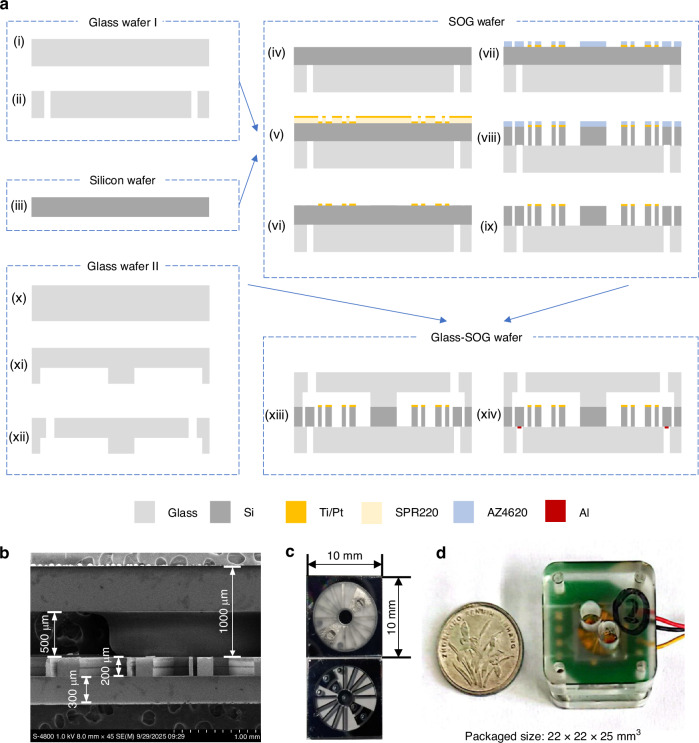
Fabrication of the MEMS-based EAA with plate-type electrodes. **a** MEMS process flow chart; **b** SEM image of the sensor cross section; **c** Pictures of the MEMS-based EAA after dicing with a size of 10 × 10 mm^2^; **d** Image of the assembled EAA comparing with a dime

To realize a miniaturized, high-performance EAA, a robust three-layer micro-electromechanical systems (MEMS) fabrication protocol was developed. This structure, illustrated in Fig. 3a, comprises a central silicon-based electrode layer sandwiched between upper and lower insulating glass substrates^28^. Specifically, to accommodate the intricate geometry of the plate-type electrodes while adhering to the aspect-ratio constraints inherent in deep reactive ion etching (DRIE), a p-type silicon wafer with a thickness of 200 μm was selected as the structural substrate. This central layer is integrated with the glass substrates through a precise two-stage anodic bonding process, ensuring both mechanical integrity and hermetic sealing.

The fabrication process follows a meticulous sequence of steps, commencing with the cleaning of the p-type silicon wafer (resistivity of 0.0005 Ω·cm) in a heated H_2_SO_4_ solution to eliminate organic residues. Concurrently, a 200 μm-thick BF33 glass wafer is laser-machined to define the through-glass vias (TGVs), after which the silicon and glass are anodically bonded to form the initial Glass-on-Silicon (GOS) stack.

Following this, a composite metal film Ti/Pt (30/150 nm) was sputtered onto the silicon surface of the GOS stack and precisely patterned to define the electrode contacts. Using AZ4620 photoresist as a mask, the silicon layer then underwent DRIE, defining the requisite flow cavities and the features of the plate-type electrodes down to the underlying glass interface. To complete the stack, a second, thicker BF33 glass wafer (1 mm-thick) is prepared through sandblasting to fabricate the fluidic reservoirs, including 1mm-diameter through-holes and a 0.5 mm-deep annular cavity, and is then anodically bonded to the processed GOS substrate. Finally, aluminum (1 μm) is evaporated into the TGVs to establish vertical electrical interconnects between the electrodes and the external circuitry.

The cross-sectional view of the Scanning Electron Microscope (SEM), presented in Fig. 3b, corroborates the successful realization of the stratified design. The image clearly delineates the functional roles of each stratum. The upper grooved glass accommodates the inertial fluid mass (electrolyte reservoir) while the basal glass layer provides structural support and robust electrical feedthroughs through the TGVs and the intermediate silicon layer houses the deep-etched cavities with metallized sidewalls, which function as the sensing plate-type electrodes. Figure 3c depicts the front and back of the diced integrated device, which features a compact footprint of 10 × 10 × 1.5 mm^3^. The electrolyte solution was vacuum-degassed prior to filling, and then injected through a pre-drilled micro-hole in the glass cover. Following this step, the chip was encapsulated with a Parylene thin film and polyethylene sealant to ensure long-term hermeticity, and further protected by a secondary-machined PMMA housing (22 × 22 × 25 mm³), as shown in Fig. 3d.

## Results and discussion

### Open-loop experiments of EAA as observation output

To verify the performance of the proposed MEMS EAA, systematic characterizations and Turntable tests were conducted, and the results are analyzed and discussed in the following sections.

#### Performance testing of EAA

Performance testing of the developed MEMS EAA was performed on a national-standard angular vibration turntable in the laboratory, with the amplitude (0–9 rad/s²) and frequency (0.01–10 Hz) precisely controlled.

**Fig. 4:**
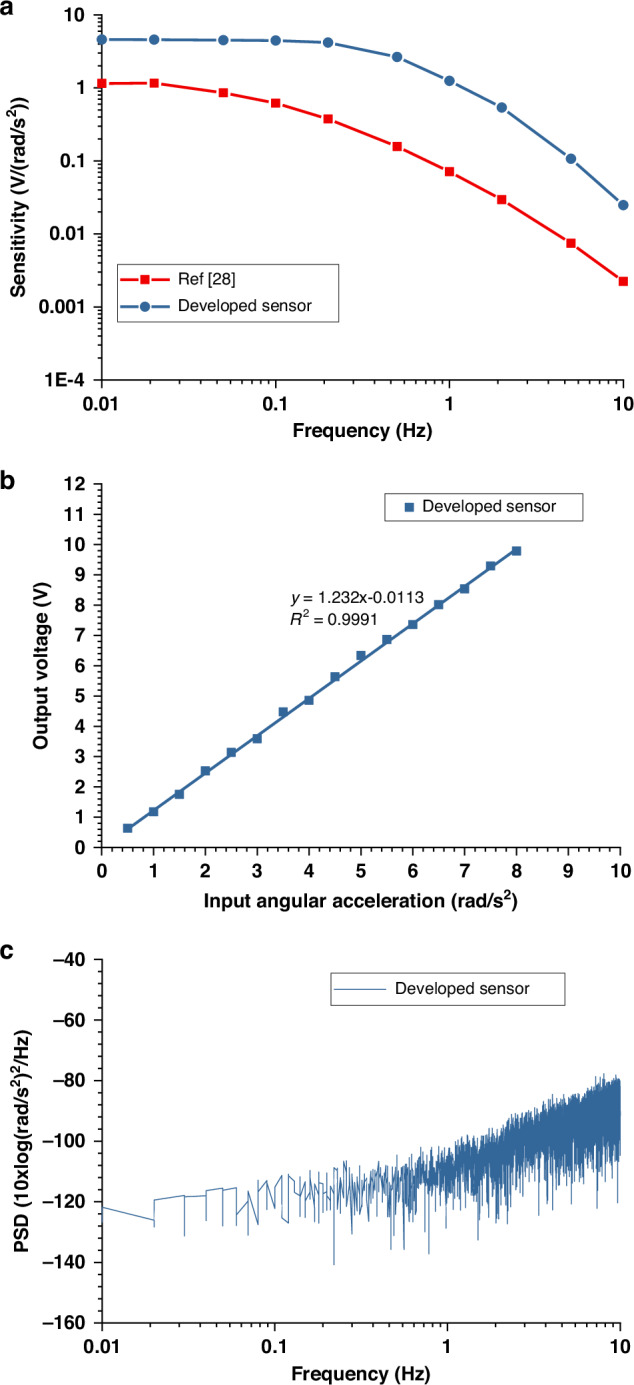
Output characteristics. **a** Amplitude–frequency response curves of the developed EAA in this work and ref. ^28^; **b** Linearity testing curve; **c** Noise level

As shown in Fig. 4a, the frequency response of the EAA was measured by recording the output voltage under varying input angular acceleration frequencies. A peak sensitivity of 4.5 V/(rad/s²) was achieved at 0.01 Hz, calculated as the output voltage divided by the input acceleration amplitude. Thanks to the increased liquid mass and the transition from conventional planar electrodes to the optimized 3D plate-type electrode architecture, the device exhibits both higher sensitivity and a wider bandwidth of 0.01–0.2 Hz (see Table 3).

**Table 3:** Performance comparison among this work and other counterparts

Electrode category	Chip size (mm^3^)	Sensitivity (V/(rad/s²))	Bandwidth (Hz)	Noise level ((rad/s²)/√Hz)	Power consumption (mW)
**Gauze-based**^29^	Ф9 × 7	0.5	\	10^−4^	16.8
**MEMS -based**^28^	10 × 10 × 1.5	1.1	0.01–0.02	7.94 × 10^−6^	4.8
**This work**	10 × 10 × 1.5	4.5	0.01–0.2	3.12 × 10^−6^	2.4

The linearity of the EAA was evaluated by measuring the output voltage against input acceleration amplitudes at 1 Hz. Linear regression in Fig. 4b gives an *R*² value of 0.998, indicating excellent linearity. Moreover, the noise performance, represented by the power spectral density in Fig. 4c, shows a noise level of 3.12 × 10^−6^ (rad/s²)/√Hz at 1 Hz. This improvement is primarily attributed to the elimination of redundant silicon areas outside the active electrode zones through DRIE. This process effectively reduces the parasitic parallel conductance and the ineffective gold-semiconductor contact area, thereby suppressing the background noise floor. Furthermore, the EAA demonstrates high energy efficiency with a total power consumption of only 2.4 mW.

In summary, compared to the prior EAA, this device offers broader bandwidth, higher sensitivity, and lower noise, while maintaining compact size and excellent linearity, making it highly suitable for UAV attitude detection.

#### Sensor regulation and experimental setup

**Fig. 5:**
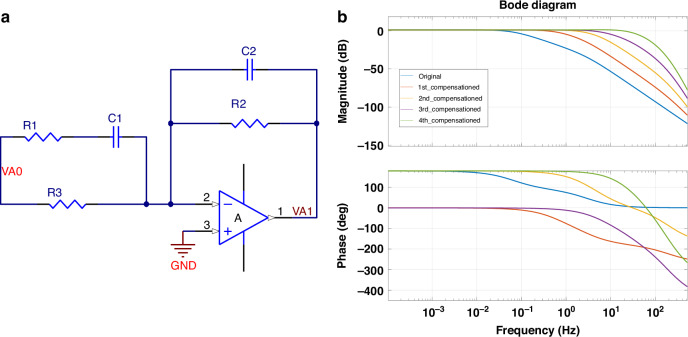
Compensation circuit design and bode plots of the EAA. **a** Compensation circuit schematic; **b** Bode plots illustrating system magnitude and phase changes before and after multi-order compensation

To further improve the high-frequency performance, a compensation circuit is introduced. Figure 5a illustrates the schematic diagram of the compensation circuit, which reconfigures the system’s pole-zero distribution. Concurrently, an active sensitivity regulation mechanism ensures high linearity during large-angle UAV maneuvers. Post-compensation results (Fig. 5b) confirm a usable bandwidth expansion to 10 Hz and significantly enhanced dynamic response, enabling reliable integration into high-agility UAV platforms. All experiments were conducted in a controlled indoor environment where the ambient temperature remained stable at 20°C ± 5 °C. The sensor’s sensitivity was also calibrated before each flight to ensure consistency.

**Fig. 6:**
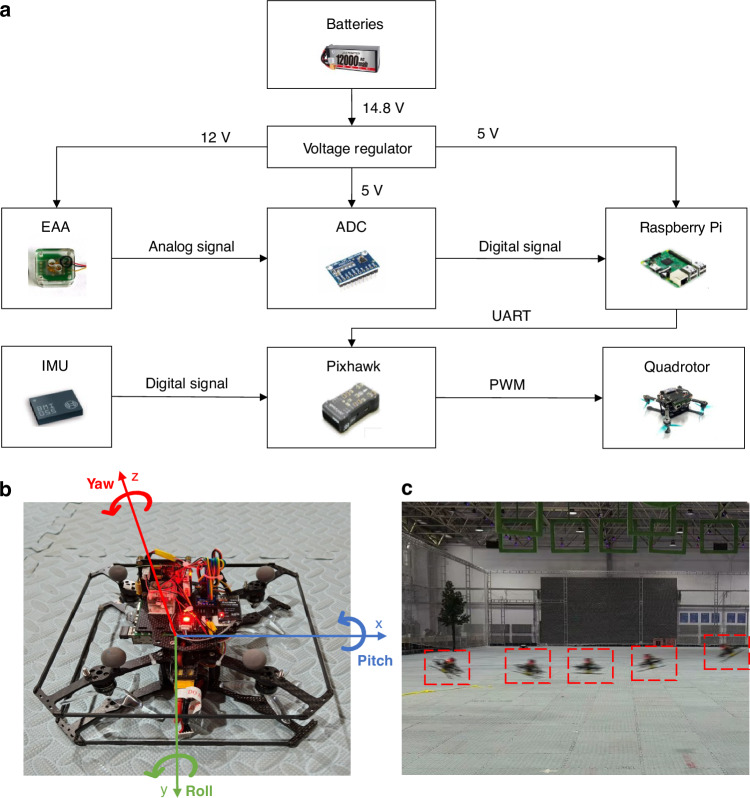
System design of the EAA-equipped quadrotor UAV. **a** Block diagram of the sensor data acquisition module. **b** Physical image of the drone equipped with the proposed EAA. **c** Remote-controlled UAV flight experiment

Depicted as Fig. 6a, the data acquisition system for the MEMS EAA-integrated UAV comprises several key modules, including a power management subsystem, signal conditioning unit, and flight control system (FCS). Power is supplied by a 14.8 V Li–Po battery, regulated down to 12 V (for analog components, including the EAA) and 5 V (for digital systems) through efficient switching converters, ensuring stable operation under dynamic conditions. The EAA’s analog output is digitized by a 32-bit ADC (ADS1263) and processed by a Raspberry Pi 4B, which ultimately transmits data to the FCS through a low-latency serial interface (TELEM1) with a sampling frequency of 250 Hz. The FCS incorporates a commercial MEMS gyroscope (BMI055) for reference angular velocity measurements, enabling cross-validation with direct EAA-derived acceleration signals to enhance attitude estimation robustness and accuracy. This integrated system supports advanced control algorithms (e.g., PID and INDI) for precise maneuverability and disturbance rejection during flight.

#### System reliability testing on turntable

Prior to UAV flight tests, system reliability testing was performed on a turntable to evaluate the dynamic response consistency between the EAA and gyroscope under controlled conditions, aiming to ensure the output reliability of the MEMS EAA integrated into a quadrotor UAV system. Specifically, the EAA is coaxially installed on the quadrotor UAV during operation.

The quadrotor UAV equipped with the EAA sensor was rigidly mounted on a single-axis rotary table to eliminate external vibration interference. The output signals of both the EAA and the gyroscope were synchronously acquired and processed through identical second-order Butterworth low-pass filters (cut-off frequency of 30 Hz). Subsequently, numerical differentiation was applied exclusively to the filtered gyroscope signal to obtain angular acceleration estimates.

**Fig. 7:**
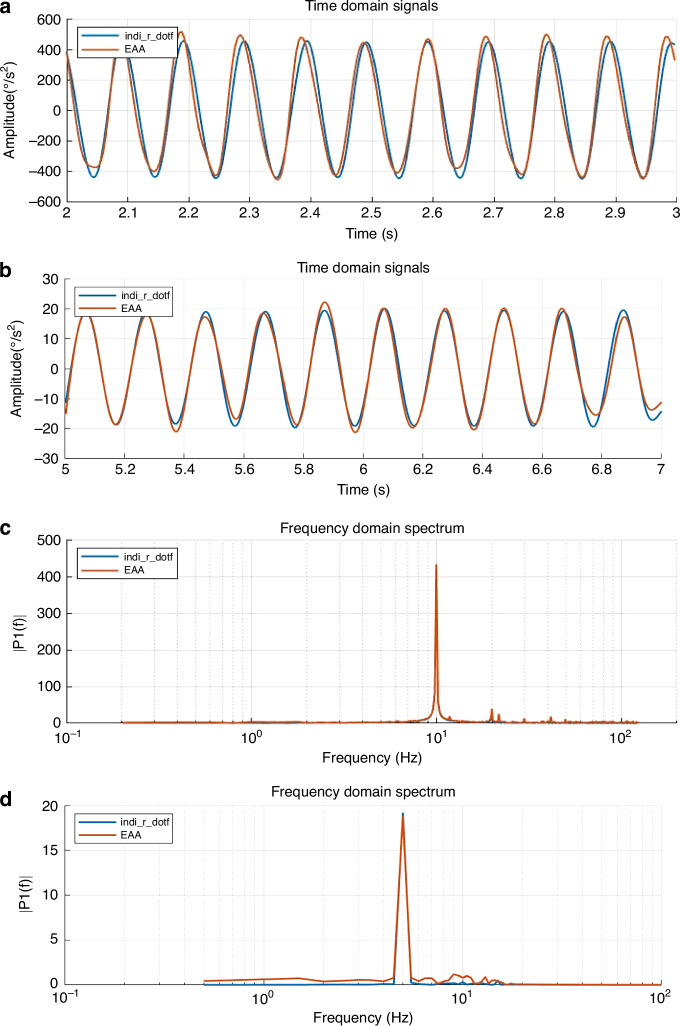
Time-domain and frequency-domain comparisons of the EAA and differentiated gyroscope signals. **a**, **b** Time-domain waveforms at 10 Hz and 5 Hz (blue: differentiated gyroscope; orange: EAA); **c**, **d** Corresponding frequency-domain spectra at 10 Hz and 5 Hz

Figure 7 shows the time-domain output waveforms and PSD of the EAA and the differentiated gyroscope signals at typical frequency points (10 Hz and 5 Hz). Time-domain comparisons (Table 4) reveal that the EAA output closely matches the differentiated gyroscope signal, with a signal consistency exceeding 0.97. A critical finding is the EAA’s significant time advantage (e.g., 12 ms at 10 Hz), which translates directly into an increased phase margin for the control system, potentially enhancing stability during aggressive maneuvers. This advantage stems from the direct measurement principle, circumventing the inherent phase lag of numerical differentiation.

**Table 4:** Consistency coefficient and time advantage of EAA compared to gyroscope

Frequency (Hz)	Consistency coefficient	Time advantage (ms)
10	0.9700	12
5	0.9732	8

This design employs standardized excitation conditions to validate the sensor’s performance consistency under simulated flight dynamics. By using controlled signals, it eliminates the influence of uncontrollable disturbances present in real flight, thereby establishing a reproducible benchmark for sensor evaluation.

### Close-loop experiments of EAA as control input for INDI system

Building upon the validated dynamic consistency and low-latency characteristics demonstrated in the turntable experiments, the subsequent phase involved integrating the EAA into the UAV’s flight control loop. To verify the EAA’s applicability in closed-loop control, two sets of experiments (step response test and hovering flight test) were conducted, with details as follows.

#### Step response test

**Fig. 8:**
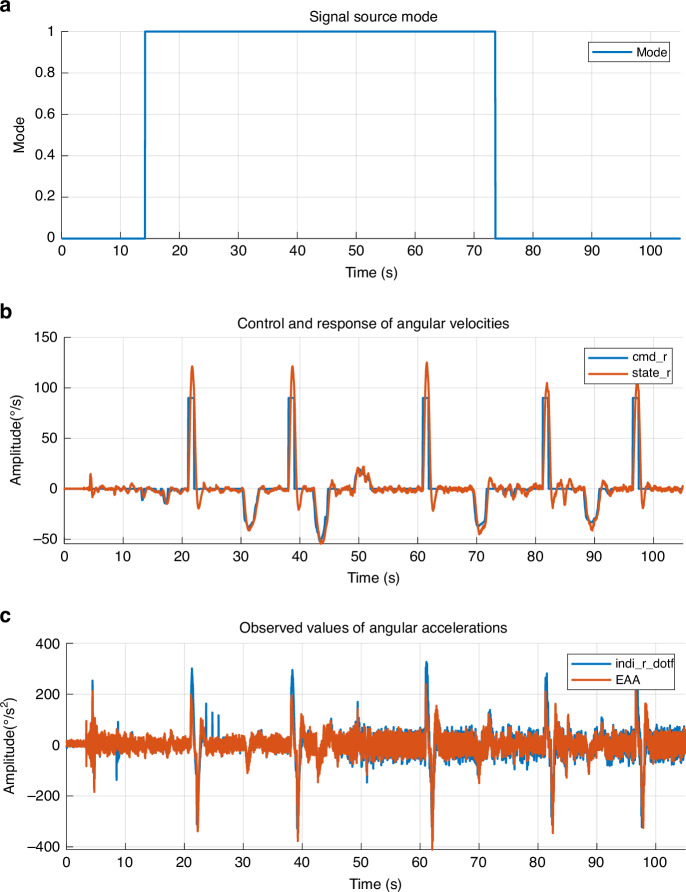
Angular velocity and acceleration responses under step excitation. **a** Signal source mode defining the active input period. **b** Comparison of commanded (cmd_r) and observed (state_r) angular velocities. **c** Angular acceleration signals from the differentiated gyroscope (indi_r_dotf) and the proposed EAA

To confirm the adaptability of the EAA as a control input for the INDI system, the EAA sensor was installed along the yaw axis of the UAV. The INDI control system was configured with a signal source switching function for the yaw channel: the Gyroscope-feedback Mode (Mode = 0) and the EAA-feedback Mode (Mode = 1). A step command (cmd_r) was sent to the UAV’s yaw channel, and the system response (state_r) was recorded. The control response data of the yaw channel after integrating the EAA into the INDI control system are shown in Fig. 8, where the Gyroscope-feedback Mode (before 14.172 s and after 73.64 s) corresponds to the gyroscope signal and EAA-feedback Mode (from 14.172 s to 73.64 s) corresponds to the EAA signal. Key performance indicators, including rise time, peak time, settling time, and overshoot, are listed in Table 5.

**Table 5:** Control metrics with EAA input

Performance metric	Gyroscope-feedback mode	EAA-feedback mode
Overshoot (%)	16.07	34.72
Rise Time (s)	0.48	0.3
Peak Time (s)	0.75	0.7
Settling Time (s)	3.73	2.4

The results demonstrate the following:

1. Step Response Characteristics: After integrating the EAA into the INDI control loop, the system exhibited an increase in overshoot (34.72% vs. 16.07%), which is primarily attributed to the direct replacement of the feedback source without re-tuning the control gains. Since the original parameters were calibrated for the high-latency gyroscope, the EAA’s 8–12 ms phase lead causes the controller to respond more aggressively. Despite this, the EAA-feedback mode achieved a significantly faster rise time (0.3 s vs. 0.48 s) and a shorter settling time, confirming its potential for high-agility maneuvers once the control law is specifically optimized for its low-latency characteristics.
2. Signal Consistency: The consistency coefficients between the control signal and the command signal were satisfactory both Gyroscope-feedback Mode (0.9453) and EAA-feedback Mode (0.9012), indicating that the EAA measurement signal can provide reliable input data for the INDI control algorithm, effectively replacing the gyroscope-derived signal for attitude control.
3. Control Performance: Overall, the integration of the EAA into the INDI control system ensured that the control accuracy and stability of the UAV’s yaw channel met flight requirements, validating the practicality of the EAA in closed-loop control applications for UAVs.

#### Hovering flight test

**Fig. 9:**
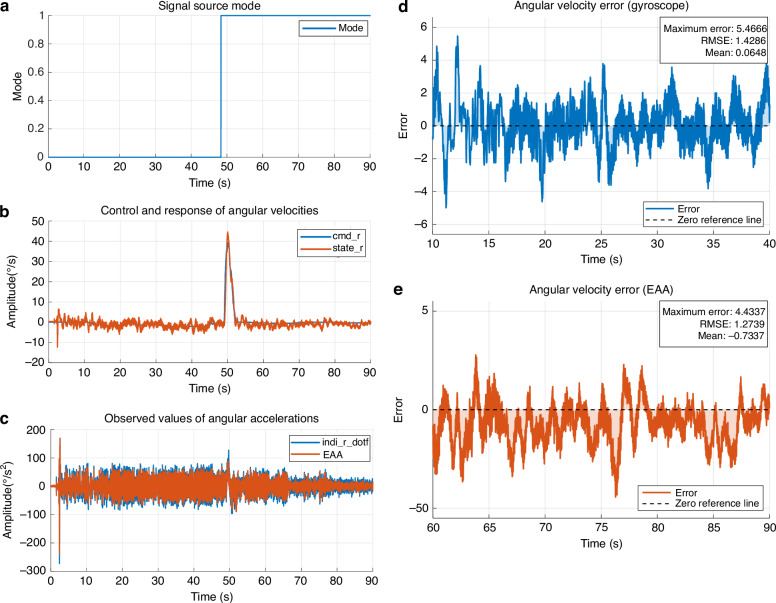
UAV hovering control and sensor error analysis. **a** Signal source mode defining the active input period. **b** Comparison of commanded (cmd_r) and observed (state_r) angular velocities. **c** Angular acceleration signals from the differentiated gyroscope (indi_r_dotf) and the proposed EAA. **d**, **e** Yaw angular velocity error curves during hovering, comparing gyroscope and EAA measurements with key error statistics

To validate the measurement accuracy, noise characteristics, and consistency with gyroscope-derived signals of the EAA under small-angle slow attitude variations during UAV hovering, the EAA sensor was installed along the yaw axis of the UAV in this study. The UAV’s motor speeds were adjusted through remote control to balance the counter-torques between motors, achieving a level hovering state with yaw angle variations constrained within ±5° (as shown in Fig. 9b). Additionally, the configuration of the UAV’s INDI control system remained consistent with the step response test settings.

Figure 9a illustrates the signal sources: gyroscope signals were used as Gyroscope-feedback Mode (before 48.3 s), and EAA measurement signals were employed as EAA-feedback Mode (after 48.3 s). Under hovering conditions, the comparison between the EAA-measured yaw angular acceleration signal (red line) and the gyroscope-derived filtered signal is presented in Fig. 9c. Key state parameters during hovering, including the root mean square error (RMSE) of yaw angular velocity and maximum angular velocity, are summarized in Table 6, which demonstrate that the method using EAA as the input exhibits superior stability. The RMSE values of the two input modes over time are 5.4666 °/s and 4.4337 °/s, respectively.

**Table 6:** Hovering performance statistics

Performance metric	Gyroscope-feedback mode	EAA-feedback mode
Angular velocity RMSE (**°**/s)	1.4286	1.2739
Maximum angular velocity error (**°**/s)	5.4666	4.4337

The experimental results indicate the excellent stability of the EAA in low-frequency, small-amplitude attitude variation scenarios, enabling precise capture of small angular acceleration changes during hovering and meeting the accuracy requirements for UAV hovering attitude monitoring.

## Conclusion

In conclusion, this study has successfully demonstrated a novel MEMS-based EAA featuring a high-performance plate-type electrode structure. By focusing on single-axis yaw stabilization as a proof of concept, this research substantiates a direct sensing paradigm for rotorcraft. The synergistic design of the fluidic channel and electrodes, coupled with a robust triple-stack bonding process, successfully achieves a compact form factor without compromising the exceptional low-frequency sensitivity inherent to electrochemical sensing. Experimental characterization, corroborated by finite element simulations, confirms that the optimized fluidic channel design effectively enhances hydrodynamic resistance, leading to superior sensitivity (4.5 V/(rad/s²)) and extended original bandwidth (0.01–0.2 Hz). Furthermore, the integration of frequency compensation circuit expands the operational to 0.01–10 Hz, validating the sensor’s capability to resolve the dynamic attitude variations required for rotorcraft stability. Beyond component-level validation, a key contribution of this work is the seamless system-level integration of the EAA within a closed-loop INDI control architecture through the direct replacement of differentiated gyroscope feedback. The sensor realized precise, real-time angular acceleration detection across diverse flight conditions, facilitating stable hovering and robust trajectory tracking. This research establishes a foundational step toward direct, low-latency inertial sensing paradigms, with broad implications for the development of next-generation high-precision autonomous aerial and robotic systems.

## Supplementary information

Support Information

## References

[1] Rieke, M. et al. High-precision positioning and real-time data processing of UAV systems. *Int. Arch. Photogramm. Remote Sens. Spat. Inf. Sci.* **38**, 119–124 (2012).

[2] Wu, H., Wang, W., Wang, T. & Suzuki, S. Sliding mode control approach for vision-based high-precision unmanned aerial vehicle landing system under disturbances. *Drones* **9**, 3 (2025).

[3] Raj, N., Banavar, R. N. & Kothari, M. Robust attitude tracking for aerobatic helicopters: a geometric approach. *IEEE Trans. Control Syst. Technol.* **29**, 150–164 (2020).

[4] Lee, T., Leok, M., & McClamroch, N. H. Time optimal attitude control for a rigid body. In *Proc. American Control Conference* 2820–2825 (IEEE, 2008).

[5] Franchi, A. et al. Full-pose tracking control for aerial robotic systems with laterally bounded input force. *IEEE Trans. Robot.* **34**, 534–541 (2018).

[6] Mozafari, S. et al. Analysis of IMU rotation effects on inertial navigation system errors. *Navigation* **72**, navi.680 (2025).

[7] Bhan, R. K., & Chaujar, R. Design optimization of MEMS gyroscope for enhanced sensitivity, bandwidth and noise reduction. *Micro Nanostruct*. **206**, 208224 (2025).

[8] Jeong, H. et al. Angular acceleration estimation with off-CG accelerometers for incremental nonlinear dynamic inversion control. *In Proc. AIAA SciTech Forum, Paper AIAA* 2024–2566 (AIAA (American Institute of Aeronautics and Astronautics), 2024).

[9] Ren, Z. et al. Enhanced attitude control of unmanned aerial vehicles based on virtual angular accelerometer. *IEEE Access* **7**, 104330–104343 (2019).

[10] Zhang, Q. et al. Incremental nonlinear dynamic inversion control for quadrotor UAV with an angular accelerometer. In *Proc. 42nd Chinese Control Conference (CCC)* 657–662 (IEEE, 2023).

[11] Nusbaum, U., Rusnak, I. & Klein, I. Angular accelerometer-based inertial navigation system. *Navigation* **66**, 681–693 (2019).

[12] Devoe, D. L. & Pisano, A. P. Surface micromachined piezoelectric accelerometers (PiXLs). *J. Microelectromech. Syst.* **10**, 180–186 (2002).

[13] Liu, H. & Pike, W. T. A micromachined angular-acceleration sensor for geophysical applications. *Appl. Phys. Lett.* **109**, 173501 (2016). Art. no.

[14] Wolfaardt, H. J. & Heyns, P. S. Dynamic modeling of a novel microfluidic channel angular accelerometer. *J. Vib. Control* **14**, 451–465 (2008).

[15] Cheng, S. et al. Dynamic fluid in a porous transducer-based angular accelerometer. *Sensors* **17**, 416 (2017). 28230793 10.3390/s17020416 PMC5336006

[16] Fu, M., Cheng, S., Wang, M., Ming, L. & Wang, T. Permeability modeling for porous transducer of liquid-circular angular accelerometer. *Sens. Actuators A Phys.* **257**, 145–153 (2017).

[17] Wang, M. et al. Modeling and simulation of the fluidic system in liquid-circular angular accelerometer based on mass-spring-damper system. In *Proc. Modeling and Simulation Conferences* (IEEE, 2018).

[18] Zeng, H. & Zhao, Y. Liquid-state motion sensing. *Sens. Actuators B Chem.* **154**, 33–40 (2011).

[19] Zeng, H. & Zhao, Y. Dynamic behavior of a liquid marble-based accelerometer. *Appl. Phys. Lett.* 96(11) (2010). Art. no. 114104

[20] Liang, T. et al. A micromachined electrochemical angular accelerometer with highly integrated sensitive microelectrodes. *Microsyst. Nanoeng*. **8**, 100 (2022). 10.1038/s41378-022-00418-7 PMC9475033 36119376

[21] Liang, T. et al. Microelectrochemical rotational vibration sensor with SOI-based microelectrodes used for seismic monitoring. *IEEE Trans. Instrum. Meas.* **73**, 9500708 (2024).

[22] Hurd, R. M. & Jordan, W. H. The principles of the solion. *Platin. Met. Rev.* **4**, 42–47 (1960).

[23] Xu, C. et al. Temperature compensation of the MEMS-based electrochemical seismic sensors. *Micromachines* **12**, 387 (2021). 33918243 10.3390/mi12040387 PMC8066024

[24] Collette, C. et al. Inertial sensors for low-frequency seismic vibration measurement. *Bull. Seismol. Soc. Am.* **102**, 1289–1300 (2012).

[25] Bard, A., & Faulkner, L. *Electrochemical Methods: Fundamentals and Applications* 2nd edn (Wiley, 2001).

[26] Kozlov, V. A. & Safonov, M. V. Dynamic characteristic of an electrochemical cell with gauze electrodes in convective diffusion conditions. *Russ. J. Electrochem.* **40**, 460–462 (2004).

[27] Zhu, M. et al. A miniaturized electrochemical angular accelerometer with the integration of microelectrodes and channel. *J. Phys. Conf. Ser.* **2740**, 012053 (2024).

[28] Zhu, M. et al. Chip-level packaged electrochemical angular accelerometer with a wide measurement range. *IEEE Sens. J.* **25**, 18941–18951 (2025).

[29] Egorov, E., Agafonov, V., Avdyukhina, S. & Borisov, S. Angular molecular-electronic sensor with negative magnetohydrodynamic feedback. *Sensors* **18**, 245 (2018). 29337874 10.3390/s18010245 PMC5795633

