# Supplementary material for: MEMS electrochemical angular accelerometer: a paradigm shift for attitude detection and control in rotorcraft UAVs
Source: Microsyst Nanoeng. 2026 Jun 4;12:217. doi: 10.1038/s41378-026-01326-w (PMC13237282; doi:10.1038/s41378-026-01326-w)
Supplement: Supplementary file 1 — Support Information [file 41378_2026_1326_MOESM1_ESM.docx]

**Supplementary Information**

**MEMS Electrochemical Angular Accelerometer: A Paradigm Shift for Attitude Detection and Control in** **Rotorcraft UAVs**

Maoqi Zhu^1,2^, Qinghua Liu^1,2^, Honghao Zhang^1,2^, Jiesong Yang^3^, Lintao Hu^1,2^, Wenlang Zhao^1,2^, Hongmin Jiang^1, 2^, Xiaoye Huo^1^, Yulan Lu^1^, Jian Chen^1,2^, Yingxun Wang^3^, Deyong Chen^1,2^, Junbo Wang^1,2^

^1^State Key Laboratory of Transducer Technology, Aerospace Information Research Institute, Chinese Academy of Sciences, Beijing 100190, China

^2^School of Electronic, Electrical and Communication Engineering, University of Chinese Academy of Sciences, Beijing 100049, China

^3^School of Automation Science and Electrical Engineering, Beihang University, Beijing 100191, China

**Corresponding Authors**

Junbo Wang, E-mail: jbwang@mail.ie.ac.cn

Deyong Chen, E-mail: dychen@mail.ie.ac.cn

Yulan Lu, E-mail: [luyl@aircas.ac.cn](mailto:luyl@aircas.ac.cn)

1. **Isolation Mechanism between Metal Interconnects and Electrolyte**

To ensure long-term electrical integrity and chemical compatibility, the Glass-on-Silicon (GOS) structure of EAA utilizes a low-resistance silicon layer as a functional interlayer to achieve isolation at the physical level. Specifically, the aluminum metallization layer is deposited within the Through Glass Via (TGV) of the bottom substrate, establishing electrical contact only with the bottom surface of the silicon layer. In contrast, the corrosive iodide/iodine electrolyte is sealed within a cavity above the silicon layer, which is formed through anodic bonding of the upper glass and silicon. The aluminum and electrolyte are physically separated by a 200-micrometer-thick silicon substrate, as signal transmission relies entirely on the bulk conductivity of the low-resistance silicon. By utilizing the silicon layer for vertical interconnection, the device ensures long-term operational reliability in a chemically active environment while maintaining good electrical contact.


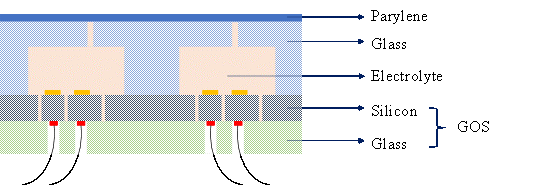


**Fig. S1 Schematic diagram of EAA profile and lead**

1. **Cyclic voltammetry Test**

Cyclic voltammetry (CV) characteristic of the EAA was characterized using a CHI660E electrochemical workstation. The potential window was scanned from -0.5V~0.5V at a scan rate of 1V/s. The resulting curve exhibits well-defined redox peaks, demonstrating the high electrochemical activity and excellent reversibility of the fabricated microelectrodes. At a bias potential of 0.3V, the output current enters a stable limiting current region, establishing a consistent baseline for dynamic signal detection. Consequently, 0.3V was selected as the optimal operating voltage while maintaining ultra-low power consumption.

**Fig. S2. Cyclic voltammetry curve of EAA.**

1. **Circuit Compensation and Noise Level ^[1]^**

The compensation strategy is established by determining the pole distribution through a fitted sensitivity curve, which serves as the foundation for reconfiguring the system's frequency response. To achieve bandwidth extension, a fourth-order lead-lag compensation circuit was implemented using low-noise OP497 operational amplifiers. The circuit applies a segmented response where the attenuation rate is reduced by 20 dB/dec across the mid-frequency band to counteract the sensor's inherent first-order roll-off, while increasing attenuation beyond 10Hz to suppress out-of-band interference. The hardware realization is achieved by calculating specific resistor and capacitor values for the impedances $Z_{1}$ and $Z_{2}$ according to the designated transfer function $H(s)=-\frac{Z_{2}}{Z_{1}}$.

In this study, the pole distribution was determined based on the fitted sensitivity curve of the raw EAA signal. To achieve the target bandwidth extension, a fourth-order compensation circuit was implemented to reconfigure the system's frequency response. The circuit utilizes the OP497 operational amplifier for its low-noise and high-precision characteristics, and the specific component parameters are summarized in Table S1.


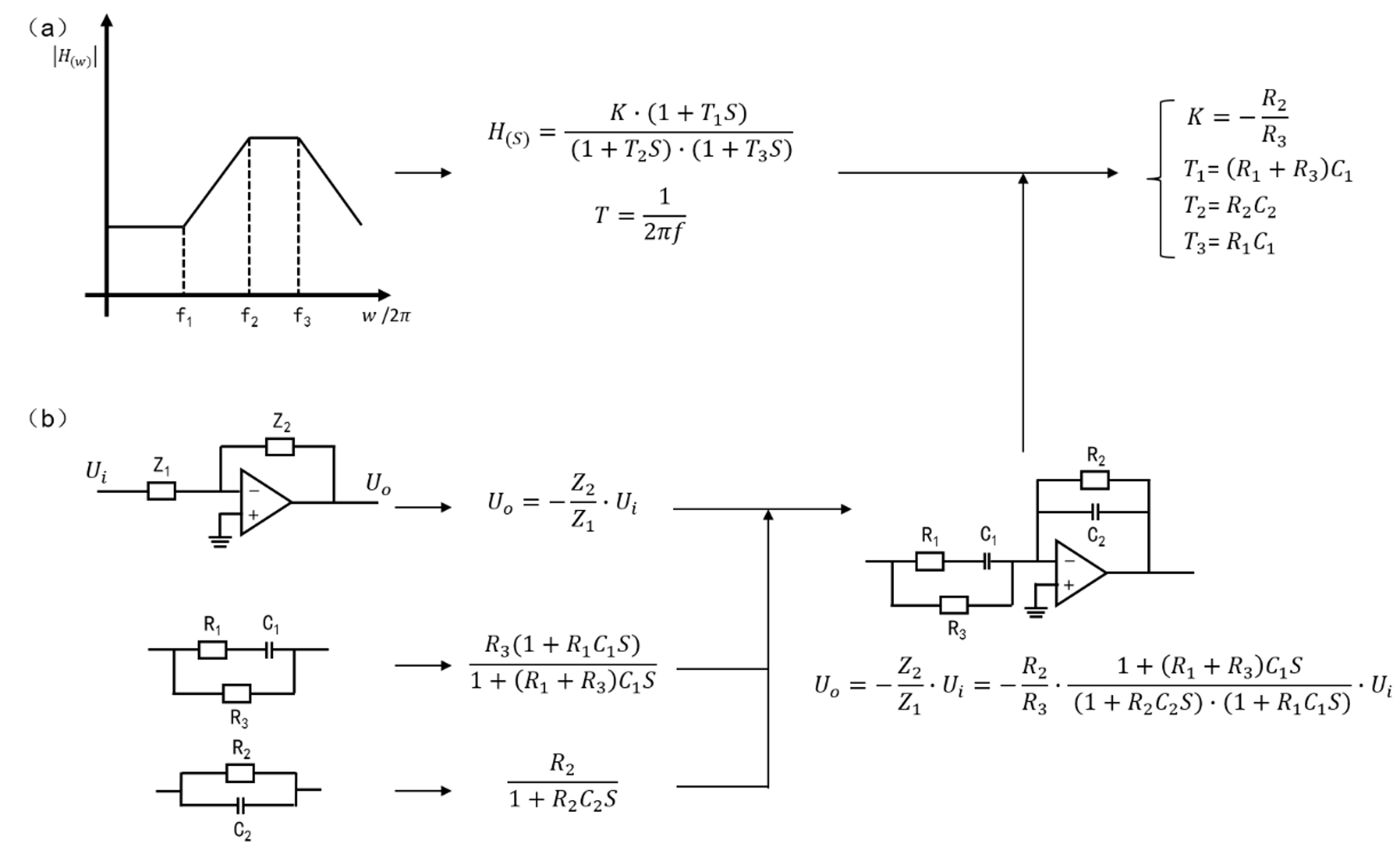


**Fig S3.** **Design scheme of frequency compensation circuit (a)** Design of transfer function; **(b)** Design of circuit structure.

Table S1 High-frequency compensation circuit component parameters

| Electronic components | R_1_/Ω | R_2_/ Ω | R_3_/ kΩ | C_1_/ μf | C_2_/ nf |
| --- | --- | --- | --- | --- | --- |
| 1st circuit | 36k | 1M | 1M | 2.2 | 220 |
| 2nd circuit | 36k | 100K | 100K | 2.2 | 220 |
| 3rd circuit | 36k | 24K | 24K | 2.2 | 220 |
| 4th circuit | 36k | 12K | 12K | 2.2 | 220 |

As shown in Fig. S4, the noise level of the device after circuit compensation is higher than its original noise level. This is attributed to the noise introduced by the compensation circuit itself, which not only amplifies the sensitivity within the frequency band compensation range but also increases the device’s noise level. For the EAA device fabricated in this paper, the self-noise levels before and after compensation are 3.12×10^-5^ rad/s²/√Hz (-110 dB) @1Hz and 8.91×10^-5^ rad/s²/√Hz (-101dB) @1Hz, respectively.

**Fig. S4 Original noise and compensated noise level of EAA**

1. **Linear Vibration Interference Test**

To verify the sensor’s immunity to structural vibration and linear acceleration of rotorcraft, we have added a linear vibration interference test in the revised manuscript. The electrochemical angular accelerometer and a reference moving-coil geophone (CDJ-Z4, sensitivity of 28 V/(m/s), bandwidth of 4-200 Hz) were fixed coaxially on a vertical vibration table. Linear vibration excitation was performed within 5-10 Hz. The linear vibration amplitude was calculated from the output of the reference geophone, and the angular acceleration was derived from the sensor output. Their ratio defines the linear vibration interference coefficient.

Test results show that the linear vibration interference coefficient of the sensor is only 6.65×10^-3^(rad/s²)/(m/s) at 5 Hz, and all values are below 1×10^-2^ (rad/s^2^)/(m/s) in 5-10 Hz. The sensor output is dominated by noise, and the interference from linear vibration is negligible, Test results show that the linear vibration interference coefficient of the sensor is only 6.65×10^-3^(rad/s²)/(m/s) at 5 Hz, and all values are below 1×10^-2^ (rad/s^2^)/(m/s) in 5-10 Hz. The sensor output is dominated by noise, and the interference from linear vibration is negligible, demonstrating excellent immunity to typical linear vibration and linear acceleration in rotorcraft environments.

Fig. S5 Time domain waveforms of geophone and EAA excited by a 5Hz vibration signal. (a) Geophone output;(b) EAA output.

Table S2 Linear vibration interference coefficient

| **Frequency/Hz** | 5 | 6 | 7 | 8 | 9 | 10 |
| --- | --- | --- | --- | --- | --- | --- |
| **Interference coefficient /(rad/s²)/(m/s)** | 6.65*10^-3^ | 8.56*10^-3^ | 4.16*10^-3^ | 5.25*10^-3^ | 8.43*10^-3^ | 7.52*10^-3^ |

1. **Cross-coupling Test**

To ensure the purity of angular acceleration measurement, a cross-coupling performance test was conducted. The EAA sensor was mounted on the turntable along the z, x, and y axes, respectively, and excited by a z-axis angular acceleration input with a frequency of 5 Hz and an amplitude of 500 °/s².

Fig. S6 (a)The z-axis main output; (b)-(c) The cross-coupling outputs from the z-axis to the x and y axes

The z-axis main output and the cross-coupling outputs from the z-axis to the x and y axes are presented in Fig. S6(a)–(c). As the measuring axis, the z-axis shows an excellent signal-to-noise ratio with a complete and clear waveform, whose peak voltage is 2.48 V. The x and y axes only exhibit distinguishable fundamental frequency components, with peak voltages of 0.04 V and 0.03 V under z-axis coupling interference, respectively. The calculated cross-coupling coefficients from the z-axis to the x-axis and y-axis are approximately 1.61% and 1.21%, respectively.

The results demonstrate that the cross-coupling interference on the non-measuring axes is extremely low. The sensor presents outstanding anti-cross-coupling performance and enables high-purity angular acceleration measurement.

1. **Random Flight Detection on UAV**

To further validate the dynamic response performance and measurement reliability of the EAA under arbitrary attitudes and large-angle rapid maneuvers of an UAV, the EAA sensor was mounted along the roll, pitch, and yaw axes of the fuselage respectively, ensuring precise alignment between the sensing axes and the UAV's body-fixed coordinate system. Throughout the experiments, the gyroscope signals were utilized as feedback inputs in control system of INDI, while the EAA outputs served solely as observational data without integration into the control loop. The UAV was operated through remote control to execute randomized flight profiles, including rapid attitude transitions within a 0°–60° range and high-dynamic maneuvers.

Fig. S7 present comparative plots of EAA-measured signals against gyroscope-derived angular acceleration (obtained through differentiation and filtering) for the three axes, respectively, with random angular accelerations reaching peak value of approximate 2000 °/s². The consistency coefficients calculated for all three axes are summarized in Table S3. Specifically, the yaw axis showed a consistency correlation coefficient of 0.9160 while the roll and pitch axis demonstrated a consistency coefficient of 0.7425 and 0.8084, respectively.

The experimental results lead to the conclusion that the EAA measurements maintained high agreement with the gyroscope-derived signals, with consistency coefficients exceeding 0.7 across all axes, under complex conditions involving large-angle and rapid attitude variation. This indicates the EAA’s exceptional dynamic response capability in accurately tracking rapid angular acceleration changes during UAV maneuvers.

**Fig. S7. Real-time axial angular velocity or angle command and corresponding angular acceleration signals measured by the EAA and the gyroscope during triaxial representative flight tests.**

Table S3 Performance Metric in 3 axes

| Axial | Yaw(r) | Roll(p) | Pitch(q) |
| --- | --- | --- | --- |
| Consistency Coefficient | 0.9160 | 0.7425 | 0.8084 |

1. **Large-Angle Flight Attitude Control Test**

To evaluate the closed-loop control performance and the feasibility of utilizing EAA as a direct feedback source during high-maneuverability flight tasks of UAV, a series of large-angle flight attitude control experiments were designed and conducted. The UAV was operated through remote control to execute randomized flight trajectories, with rapid attitude commands ranging from 0° to 60° sent to its pitch axis. Fig. S8(a) illustrates the switching interval of angular acceleration sources during a representative flight: the EAA signal served as the control input during the 10–60 s period, while the IMU-derived signal was employed from 62 to 95 s. The corresponding pitch angle command (cmd_theta) and pitch angle response (state_theta) are presented in Fig. S8(b). Fig. S8(c) and (d) depict the system’s angle control errors computed using the EAA and IMU as angular acceleration sources, respectively, with detailed results summarized in Table S4. For the pitch axis, the root mean square error (RMSE) of angle tracking was 7.7864° with a maximum error of 29.4167° when utilizing the EAA input, whereas the RMSE and maximum error reached 8.8593° and 37.4501° under IMU input conditions. Experimental results demonstrate that under high-dynamic conditions such as large-angle flight, employing the direct angular acceleration signal from the EAA as control feedback not only ensures robust system stability but also achieves superior angle tracking accuracy compared to conventional IMU differential signals.

**Fig. S8.** **Comparison of pitch control performance using EAA and IMU in large-angle flight attitude control test.** **(a)** Switching sequence of angular acceleration control sources;**(b)** Pitch angle command and response curves;**(c)** Angle tracking error under EAA-based control;**(d)** Angle tracking error under IMU-based control

Table S4 Performance Metric in 3 axes

| **Performance Metric** | **Gyroscope-feedback Mode** | **EAA-feedback Mode** |
| --- | --- | --- |
| **Angle velocity RMSE (°)** | 8.5398 | 7.7864 |
| **Maximum angle error (°)** | 37.4501 | 29.4167 |

1. **INDI Control Strategy ^[2]^**

(**1**) **General INDI control law**

The basic description of the affine nonlinear system is given by:

Where $x \in R^{n}$is system state vector; $u \in R^{p}$ is system control input vector; $f(x,u)$ is state transition function of the nonlinear system; is first derivative of the state vector (rate of state change).

Through the first-order Taylor expansion and neglecting the high-order small quantities of state increments (the principle of time-scale separation), the incrementally linearized model is obtained:

Where $x_{0}$ is the system state vector at the previous sampling moment; $u_{0}$ is the system control input vector at the previous sampling moment; is the rate of state change at the previous sampling moment; $G(x_{0},u_{0})=\frac{\partial f(x,u)}{\partial u}|_{x=x_{0},u=u_{0}}$is input Jacobian matrix, reflecting the influence of control input on the rate of state change; $\tilde{u}= u-u_{0}$ is control increment, which is the core output of INDI control.

Taking the desired rate of state change as the virtual control input , the core incremental control law is solved as:

Where $v$ is the virtual control input, i.e., the desired rate of state change; $G^{-1}(x_{0},u_{0})$ is the inverse matrix of the input Jacobian matrix $G(x_{0},u_{0})$ (suitable for full-rank systems);

In practical engineering, the final control input is the sum of the input at the previous sampling moment and the control increment:

$$u=u_{0}+\tilde{u}$$

**(2) INDI control law for quadrotor attitude loop**

Taking angular acceleration as the core feedback, the mapping relationship between the increments of angular acceleration/thrust and the rotor speed increment is derived by combining the rigid body rotation equation of the quadrotor:

Where is angular acceleration vector in the body frame, where $p,q,r$ are the roll, pitch and yaw angular rates, respectively;

is the filtered angular acceleration increment ( is the filtered angular acceleration at the previous moment);

$T$ is the total thrust of the quadrotor, and $\tilde{T}_{f}=T-T_{0}$ is the filtered thrust increment ($T_{0}$​ is the filtered thrust at the previous moment);

$G_{1}$ is the gain matrix of thrust/torque-rotor speed for the quadrotor attitude loop, determined by the thrust coefficient $C_{T}$​, torque coefficient $C_{M}$​, distance d from the rotor to the center of mass, and moment of inertia $I_{\text{x}}/I_{\text{y}}/I_{\text{z}}$;

$G_{2}$​ is the gain matrix related to the rotor gyro torque, determined by the rotor moment of inertia $I_{\text{rp}}$​, sampling time $T_{s}$​, and yaw moment of inertia $I_{\text{z}}$​;

$\text{diag}(\omega_{f})$ is the diagonal matrix formed by the filtered rotor speed vector;

$\omega=[\omega_{1},\omega_{2},\omega_{3},\omega_{4}]^{T}$is the rotor speed vector of the four rotors of the quadrotor, and $\tilde{\omega}_{f}=\omega-\omega_{f}$ is the filtered rotor speed increment ($\omega_{0}$​ is the filtered rotor speed at the previous moment);

$z^{-1}$is the unit delay operator in the discrete domain, representing the signal at the previous sampling moment;

The subscript $f$ denotes the physical quantity after filtering.

By inverting Eq. (4), the solution formula for the rotor speed increment is obtained:

Where is the desired angular acceleration of the attitude loop (virtual control input); is the filtered actual angular acceleration at the current sampling moment; $(G_{1}\text{diag}(\omega_{f})+G_{2})^{-1}$is the inverse matrix of the attitude loop gain matrix;

Among them, the virtual control input is the desired angular acceleration, generated by the PD regulation of the attitude angle/angular rate error:

Where is the actual Euler angle vector in the body frame, where are the roll, pitch and yaw angles, respectively;

$\xi_{ref}$ is the desired Euler angle vector;

$\text{Ω}=[p,q,r]^{T}$is the actual angular rate vector in the body frame;

$K_{\xi},K_{\text{Ω}}$ are diagonal positive definite gain matrices, which is the proportional/derivative gain of PD regulation for attitude angle and angular rate, respectively.

The block diagram of the quadrotor INDI cascaded flight control system with EAA/gyro dual feedback is as follows:

Fig S9 The block diagram of the quadrotor INDI cascaded flight control system with EAA/gyro dual feedback

**Reference**

1. C. Xu, J. Wang, D. Chen, et al., “Temperature compensation of the MEMS-based electrochemical seismic sensors,” *Micromachines*, vol. 12, no. 4, p. 387, 2021.
2. J. Yang, Z. Cai, J. Zhao, et al. "INDI-based aggressive quadrotor flight control with position and attitude constraints." *Robotics and Autonomous Systems,* vol. 159, p. 104292, 2023.
